# Aetiology of Myeloproliferative Neoplasms

**DOI:** 10.3390/cancers12071810

**Published:** 2020-07-06

**Authors:** Mary Frances McMullin, Lesley Ann Anderson

**Affiliations:** 1 Centre for Medical Education, Queen’s University, Belfast BT9 7AB, UK; 2 Centre for Health Data Science, University of Aberdeen, Aberdeen AB24 3FX, UK; l.anderson@qub.ac.uk

**Keywords:** epidemiology, myeloproliferative neoplasms, polycythemia vera, essential thrombocythemia, lifestyle factors, smoking

## Abstract

Myeloproliferative neoplasms (MPNs) have estimated annual incidence rates for polycythemia vera (PV), essential thrombocythemia (ET), and primary myelofibrosis of 0.84, 1.03, and 0.47 per 100,000. Prevalence is much higher, particularly for PV and ET, as mortality rates are relatively low. Patients are often concerned about why they developed an MPN and epidemiological studies enable the identification of potential causative factors. Previous work in small heterogeneous studies has identified a variety of risk factors associated with MPNs including family history of MPN, autoimmune conditions, some occupational exposures, and blood donation. At a population level, germline predisposition factors in various populations have been associated with MPNs. The pilot MOSAICC (Myeloproliferative Neoplasm: An In-depth Case-Control) study is one of the largest epidemiological studies in MPN ever carried out to date. It demonstrated the most effective methods for carrying out a significant epidemiological study in this patient group including the best way of recruiting controls, as well as how to evaluate occupational and lifestyle exposures, evaluate symptoms, and collect biological samples. Significant results linked to MPNs in the pilot study of 106 patients included smoking, obesity, and childhood socioeconomic status. The methodology is now in place for a much larger ongoing MOSAICC study which should provide further insight into the potential causes of MPNs.

## 1. Introduction

Myeloproliferative neoplasms (MPNs) are a heterogenous group of acquired clonal disorders where an abnormal haematopoetic stem cell transforms myeloid progenitors leading to overproduction of one or more types of myeloid cells. The classic MPNs are polycythemia vera (PV), where there is overproduction of predominantly erythroid cells; essential thrombocythemia (ET), where platelet overproduction is the major issue; and myelofibrosis (PMF), where megakaryocyte excess leads to increased fibrosis of the bone marrow. These disorders are referred to as Philadelphia negative MPNs distinguishing them from the Philadelphia positive disorder chronic myeloid leukaemia, which is also classified as a MPN in the 2016 revision to the WHO classification of myeloid neoplasms [1].

Since 2005, clonal abnormalities have been discovered in the classic MPNs delineating the nature of the acquired clone. The *Janus Kinase* 2 (*JAK*2) V617F mutation was discovered in 95% of patients with PV and approximately 50% of those with ET and PMF [2]. Subsequently *myeloproliferative leukaemia virus oncogene* (*MPL)* mutations and then *calreticulin* (*CALR*) mutations have been discovered in many of those who did not have a *JAK*2 mutation [3,4]. There remain a decreasing number in whom no mutation has yet been found, referred to as triple negative.

After various iterations, the most recent World Health Organisation (WHO) classification of diseases in 2016 sets out diagnostic criteria for each MPN recognising the acquired clonal nature of the diseases and therefore classifying them as neoplasms [1].

The discovery of a consistently abnormal blood count can lead to the incidental finding of an MPN, but many patients present with symptoms such as tiredness, night sweats, itching, and early satiety. Signs include bruising and splenomegaly. Excess morbidity and mortality results from thromboembolic events and PV and ET have the potential to transform to MF. All can transform to a myelodysplastic syndrome or acute leukaemia which are severely life limiting events as was seen in a population bases study performed in Sweden [5].

## 2. Incidence and Prevalence of MPNs

Incidence refers to the number of new cases of a disease diagnosed within a specified time frame. The classic MPNs are considered rare disorders according to RARECAREnet with an estimated annual incidence rate of 2.17 cases per 100,000 of the population [6]. Prevalence is the number of cases of a disease in the population at any point in time. The prevalence rate of MPN is much higher than the incidence rate. MPN patients can live with the disorder for many years with life expectancy variable by MPN subtype. A population-based study from Sweden showed that 10-year survival rates were significantly lower than the general population for PV, ET, and PMF patients. This may be due to MPN patients having a higher risk of developing major events such as serious thromboembolic disorders and development of second cancers, including acute leukaemia, which is reported in 1 in 10 MPN patients [7,8]. 

Many small studies have been undertaken to try to determine incidence and prevalence of MPN subtypes. Our research group carried out a detailed systematic review of the published literature in order to estimate the incidence of PV, ET, and PMF [9]. A systematic literature search was carried out in multiple databases from 1946 to August 2012. Thirty-four studies were included for analysis and authors were contacted where possible for additional study data for analysis. This data was investigated to produce a meta-analysis of the data available [9].

In the systematic review, 20 studies assessed incidence of PV (studies from Europe, North America, Australasia, and Asia) with a pooled annual incidence rate of 0.84 per 100,000 [9,10]. One study from Japan likely underestimated the incidence of PV due to limited country coverage and was excluded from the combined estimate [11]. Crude annual incidence rates did not differ significantly between males and females or by geographical region [9,10].

Ten studies reported incidence of ET (from North America and Europe) with a pooled annual incidence rate of 1.03 per 100,000. The incidence of ET appeared higher in Europe (1.60 per 100,000) than North America (0.96 per 100,000) and higher in males than females [9,10].

Twelve studies from Europe, North America, and Australasia reported a combined annual incidence rate of PMF of 0.47 per 100,000. No significant difference between geographical areas was observed and annual incidence rates did not differ significantly between males and females [9]. 

A small number of studies, mostly from Europe, reported incidence of the classic MPNs combined with a pooled annual incidence of 2.58 per 100,000 [9].

In the systematic review, only eight studies were identified reporting on MPN prevalence rates. Prevalence rates for PV ranged from 0.49 to 46.88 per 100, 000 and for ET from 11.00 to 42.51 per 100,000. Only two studies reported prevalence for PMF with prevalence rates of 1.76 and 4.05 per 100,000, respectively [9].

With increasing cancer registration and better diagnostics, including routine screening for the *JAK*2 mutation since 2005, many groups and countries have analysed changing MPN patterns of incidence. The Surveillance, Epidemiology, and End Results (SEER) Program in the US is a very large database which has been analysed with incidence rates of 1.09 per 100,000 for PV, 0.96 per 100,000 for ET and 0.31 per 100,000 for PMF calculated [12]. All MPNs were associated with lower incidence rates in Hispanic whites compared to non-Hispanic whites [12]. 

In Europe data was examined from publications, registries and databases. Widely varying annual incidence rates were reported. From 0.4 to 2.8 per 100,000 in PV, 0.38 to 1.7 per 100,000 in ET, and from 0.1 to 1 per 100,000 in PMF [13]. Few prevalence rates were reported and because of various reporting methods they were difficult to compare [13]. 

In Norway from 1993 to 2012, incidence of PV increased from 0.4 to 0.7 per 100,000, ET from 0.3 to 1.0 per 100,000, and PMF from 0.2 to 0.5 per 100,000 [14]. In Sweden, where cancer registration was mandatory between 2000 and 2014, the incidence of PV remained stable ranging from 1.18 to 1.75 cases per 100,000 [14]. The incidence rate of ET increased over time from 1.26 per 100,000 in 2000 to 1.94 per 100,000 in 2014. Similarly, incidence of PMF increased slightly over time [14]. The number of MPN patients who were recorded as unclassified decreased over time demonstrating an improvement in the classification of cases [15]. Conversely, in Australia, between 2003 and 2014, incidence of MPNs have been seen to decrease by −2.9% per year [16]. This was mostly driven by PV incidence and probably reflects more accurate diagnostics with the inclusion of molecular testing for *JAK*2 mutations [16].

Prevalence remains difficult to determine. In Korea, between 2004 and 2013, analysis of a comprehensive population database gave prevalence rates for PV from 2.8–5.4 per 100,000, in ET from 4.1–9.0 per 100,000, and in PMF for 0.5–0.9 per 100,000 [17]. Prevalence increased in all MPN subtypes over the period of the study [17]. However, the prevalence rate reported may be inaccurate, and may grossly underestimate the presence of these neoplasms in the population as studies have found the acquired mutations in normal populations [18]. Also, a population study from Denmark using sensitive methods found a prevalence of 3.1% for the *JAK*2V617F mutation and 0.16% for *CALR* mutations in a general population [19,20]. If the presence of acquired mutations such as *JAK*2 or *CALR* represents an MPN, then the prevalence of MPNs may be much higher than has currently been reported.

## 3. Etiological Studies: Principles and Methods

Identifying the cause (aetiology) of MPNs is important in order to determine causal mechanisms, preventative strategies and curative treatments. Observational studies enable researchers to identify associations between potential risk factors and a disease but cannot be used to determine causation. Observational studies observe patients in a non-controlled environment looking to identify differences between patients with disease and those without. A number of observational study designs are available to assess the cause of disease. These include cohort studies, case-control studies, and ecological studies. 

### 3.1. Cohort Studies

These identify a population or representative sample of a population, collect information, and follow participants over time to see who develops the disease. These studies have a number of advantages including low recall bias but are costly and need to follow a very large population if investigating potential causes of rare conditions, such as MPNs. Nested case-control studies can be conducted using data from a cohort study to identify cases of disease and a control group looking retrospectively at collated data. This approach is primarily used when investigating risk factors using healthcare data. Case-cohort studies are a more efficient method to assess potential risk factors where a random sample of the cohort and all cases are selected. 

### 3.2. Case-Control Studies

These studies identify patients with a disease and a ‘control’ group and ask participants to recall information about a range of potential risk factors. This study design is best suited to investigating the causes of rare diseases, but are subject to recall bias which can be particularly problematic if it differs between case and control groups. 

### 3.3. Ecological Studies

Studies which are used when individual patient information is not available. They can identify correlations between potential risk factors and disease but are only applicable at a population level. They are subject to a type of confounding called ecological fallacy where associations may be identified at a group level but not be true for individual patients.

## 4. Genetic Factors in the Development of MPN

However, before the epidemiology of an MPN is considered the issue of an underlying genetic disposition always arises. In some families there is an inherited gene mutation which leads to a congenital disorder such erythrocytosis (*erythropoietin receptor* mutations) or thrombocytosis (*thrombopoietin* mutations) [20,21]. In one study of MPN patients, 8% reported a family history of MPN [22]. Familial aggregation of cases may be due to inherited gene mutations and/or shared environmental and/or lifestyle exposes.

A number of germline genetic patterns have been found to be associated with patients who develop an MPN (Table 1). The *JAK*2V617F mutation arises preferentially on a specific constitutional *JAK*2 haplotype. This haplotype referred to as 46/1 or GGCC is very common occurring in 50% of the normal population. It is therefore a low penetrance predisposition allele and is estimated to account for 50% of the attributable population risk of developing a MPN [23]. 

In a genome-wide study in the Icelandic population, a common variant rs2736100-C in the second intron of the *telomerase reverse transcriptase (TERT*) gene was associated with MPNs [25]. The *TERT* variant exerts a similar risk on all three MPN subtypes and the risk is large. This association was confirmed in a large Italian cohort of sporadic MPNs [26]. These common variants at *TERT* and *JAK*2 loci account for most of the population risk of MPN. In other cohorts, using genome-wide association studies multiple germline variants have been identified which predispose to MPN [30,31]. Some of these associations (*MECOM* rs2201862, *HBS1L-MYB* rs9376092, and *THRB-RARB* rs4858647) have also been investigated in a large Romanian cohort of patients [28]. 

In addition to a genetic predisposition to develop MPN, several families have been described where a germline variant has been identified in the family which leads to a genetic predisposition to develop MPN (Table 1). In these families, the acquired MPN is no different from those in sporadic cases of MPN with the occurrence of a variety of MPNs and different acquired driver mutations in the same family with the germline variant. In total, four families from the French West Indies are described, with an adult onset haematological malignancy particularly ET, with half the cases progressing to myelofibrosis or acute myeloid leukaemia. In the four genetically related families germline copy number variation is inherited and leads to overexpression of *ATG2B* and *GSKIP* resulting in enhanced haematopoietic differentiation and increased progenitor sensitivity to thrombopoietin. This results in increased fitness for cells bearing somatic driver mutations [24]. In a single Australian family of English heritage, MPN was diagnosed over four generations, with three different driver mutations identified. This was linked to the *RBBP6* -R1569H mutation. RBBP6 (retinoblastoma binding protein6), a ring finger E3 ubiquitin ligase, which ubiquitinates and degrades p53. The mutation is located near the p53 binding site causing elevation in somatic mutagenesis [27]. In a Finnish family with four individuals with PV in two generations, exome sequencing identified novel candidate predisposition variants in the gene *LRRC3* (Leucine-rich repeat-containing 3), and a rare, predicted benign variant in *BCORL1* (BCL6 Corepressor Like1) in all patients. These novel variants were not found in other Finnish families with two cases of PV in each family [29].

In summary, there are a small number of common genetic variants associated with familial predisposition to MPN. Very rare families exist where a specific inherited gene mutation has been identified which predisposes to development of an MPN.

## 5. Epidemiological Studies in MPN

Although genetic factors predispose to the development of MPNs it is likely that environmental and/or lifestyle factors also have a role in the development of these disorders. Many such factors may be contributory and need to be considered including factors such as inflammation which may be reflected by previous illness. A small number of studies including cohort, nested case-control/case-cohort and case-control studies have been undertaken in the past. In order to try to evaluate what was known about the epidemiology of MPN in 2010, we carried out a systematic literature search using broad MPN related search terms. From an original total of 6315 articles meeting the search criteria, 19 articles were identified which assessed risk factors for the classic MPNs. There were 6 cohort and 13 case-control studies identified. These articles were analysed with a number of potential risk factors identified for MPNs (Table 2) [10].

The review identified a variety of environmental, lifestyle and familial/ethic factors associated with MPNs. However, there was significant heterogeneity in case definition, study design and in particular the risk factors investigated. Some of the studies had small sample sizes and therefore were of limited power to detect associations between potential risk factors and MPNs.

The strongest evidence for an increased risk of MPNs was in those with a family history of MPN reflecting certain genotypes associated with the development of MPNs discussed previously. Jewish descent was also strongly associated with MPNs in several studies with risk highest in those with Ashkenazi Jewish descent in a study from Northern Israel [32,33].

Cohort studies of poultry workers, commercial pressmen, petroleum refinery workers, and funeral service workers identified a higher risk of PV or PMF or an increased mortality rate from these conditions compared to the general population. In case-control studies, agricultural workers and those in rural sector jobs were at a higher risk of developing MPNs. Cooks, waiters, clerks, and those working with electrical devices were more likely than controls to have ET [10].

Benzene exposure may well be a risk factor for MPNs. Petroleum was associated with an increased risk of MPNs [34,35]. Using dark hair dye for more than 10 years was significantly associated with ET [36]. Rural residence was also associated with a higher risk of MPNs [37] as was living in a Tuff house (a house made gamma emitting volcanic porous material) for more than 9 years in ET patients [36].

Autoimmune disorders have been investigated in association with MPNs. In particular, Crohn’s disease has been identified in several studies with giant cell arteritis also significantly associated with MPNs in one study [38,39].

Of note, PV was more common than expected in a cohort of active blood donors from Southern Stockholm which was also observed in blood donors in France [32,40]. However, a more recent study of 1.4 million blood donors from Scandinavia did not find any increased risk of PV in blood donors [44]. 

Other factors may also be considered to be associated with MPNs as seen in some recent studies. In a number of populations studied some positive associations have been noted. Smoking in clinical practice is associated with higher blood counts. It is genotoxic and therefore would be considered a candidate risk factor for MPNs. In the Million Women Study, smoking was associated with a 42% increased risk of MPN/myelodysplastic disease when compared to never smoking [41]. The Iowa Woman’s Health Study found an association current smoking and MPN and PV [45]. A large population study from Denmark and meta-analysis of studies looking at blood counts, smoking, and MPN showed that smoking was associated with increased leukocyte counts and risk of MPNs in current smokers [46]. No associations between alcohol and MPN have been identified. Caffeinated coffee consumption was inversely associated with, and therefore appeared to be protective against, PV whereas there was no association with decaffeinated coffee [47].

Obesity has been associated with a number of cancers including blood cancers. An increased risk of MPN/myelodysplasia was associated with each 10 kg/m^2^ increase in body mass index in the Million Women Study, an extensively followed cohort of almost half a million participants which included 490 MPN cases [42].

Ionising radiation is another potential factor which could be associated with MPNs. A study of MPN patients who had been exposed to ionising radiation at Chernobyl were compared to a control group with MPN who had not been exposed to radiation, the pattern of driver mutations found in the exposed group was different suggesting some influence of the radiation exposure in the development of MPNs [43].

## 6. The MOSAICC Study

Epidemiological studies of MPNs have been limited by small sample sizes, inability to investigate subtype specific risks, and variability of methodological approaches utilised. In order to explore this issue further we carried out an initial exploratory case-control study of MPNs in order to determine the most feasible methodological approaches, case and control ascertainment and collection of biological samples by way of a pilot study. The MOSAICC (Myeloproliferative Neoplasms: An In-depth Case-Control) study recruited patients from two UK sites (Belfast City Hospital, Belfast and University Hospital Southampton NHS Foundation Trust, Southampton) with MPN status classified using WHO diagnostic criteria [1]. Two control groups were used, with patients asked to recruit non-blood relative or family friend (NBR/F) controls, and age and sex frequency-matched general practice (GP) controls recruited. Consenting patients and recruited controls underwent a structured telephone interview with a trained interviewer. Participants were asked to provide a saliva specimen and requested to have a blood sample taken both for DNA collection to later investigate gene-environment interactions.

Data was collected on sex, age, recruiting clinic, Jewish ancestry, childhood socioeconomic position (SEP), birth order and number of siblings, level of education, pack years of smoking, alcohol consumption, self-reported height and weight, piercings, tattoos, tooth filings, implants, hair dye use and storage of chemicals in the home, hobbies and air travel, and pre-existing medical conditions. An occupational assessment was also carried out using OccIDEAS, a platform to assess exposure to potential carcinogens.

The completion of the MOSAICC pilot study showed that it was feasible to recruit to and carry out a systematic case-control study of MPN patients. The telephone interviews and data collection were successful in accumulating a dataset of comprehensive information. In total, 106 MPN cases and 120 controls were recruited. Of the controls, 17% were GP and 72.5% were NBR/F. Despite the disadvantages of using NBR/Fs as a control group (they are more likely to be similar to cases) it was identified as a much more suitable method to recruit controls.

Despite the fact that this was designed as a pilot study, MOSAICC recruited more patients and controls than any previous case-control study of MPNs. There was therefore sufficient data to analyse medical and life style factors associated with MPN [48]. MPN patients were more likely than controls to be raised in a household where the main occupation of the household reflected lower socio-economic status [48]. Current cigarette smoking was more common in cases than controls but statistically significantly elevated for PV cases only [48]. Alcohol consumption was inversely associated with PV and ET [48]. Obesity was significantly associated with ET only and not with other MPN subtypes [48]. No significant associations were observed for hair dying, implants, piercings, or tattoos identified in previous case-control studies (Table 2) [48].

With regards environmental and other potential risk factors, the majority of cases and controls had pets but only pig ownership was significantly higher in cases compared to controls [48]. This result must be treated with caution as the actual numbers of pig owners was very small and one of the study areas has a large rural population so it may be a chance association. Painting and home decorating were less common in MPNs than in controls and no significant associations were seen with use of potential household carcinogens including home solvents, pesticides, oven cleaner, and home car mechanical work [48]. There were no differences from controls in the frequency of short or long-haul flights taken per year [48]. 

No association with MPN was seen with any preceding medical condition diagnosed [48]. Frequency of CT scans was associated with MPN, with MPN patients more likely to receive three or more CT scans than controls [48].

## 7. Other Findings from MOSAICC Study

In the MOSAICC study, patients and controls were asked to provide two biological samples, a blood sample, and a self-collected saliva sample. Of the participants, 89% had a blood sample taken and submitted and 93% submitted a saliva sample. DNA yield from both types was excellent although significantly higher from blood [49]. This study demonstrates that it is possible to obtain biological samples from the majority of participants in case-control studies, therefore building a bank for future molecular epidemiological studies with controls.

MPN patients have a severe symptom burden. MPN-SAF, a reliable and validated tool for the assessment of symptoms in MPN, was given to patients and controls in MOSAICC. The results for the UK patient group were compared to MPN-SAF results from a US group of 1446 Mayo clinic patients [50] and symptom burden was broadly similar in the two groups [51]. However, compared to the control group and MPN, patients had a significantly higher symptom score than controls in 25 out of the 26 parameters measured. Fatigue was the most common symptom. This is the first time MPN-SAF scores were assessed in a control group and this reinforces the presence of the symptom burden suffered by MPN patients [51].

During the telephone interviews MOSAICC patients were asked about their perceived diagnosis. Answers were correlated with the haematologists reported diagnosis. In a group of patients with MPN, only 77% reported that their diagnosis was MPN, 40% recognised MPN as a ‘blood condition’, and 24% as ‘cancer’. Patients’ perspectives of their diagnosis showed considerable discrepancy from the haematological diagnosis which may reflect many different patient personalities and coping strategies [52].

## 8. The Way Forward

The MOSAICC pilot study showed that it is feasible to conduct a large case-control study of MPNs and identified the most suitable methodological approaches to use. Recruitment was excellent, generating sufficient data to identify significant associations between risk factors and MPNs. However, using the proven methodologies, it is necessary to carry out a much larger multicentre study in order to generate a large enough body of data to study risks for MPN subtypes. This study is now in progress and should recruit at least 500 cases with MPNs in the United Kingdom. Biological samples will be collected, providing a resource for molecular epidemiological study of MPNs in the future.

## 9. Conclusions

Multiple risk factors have been associated with the risk of MPNs. Epidemiological studies in the past have found associations between the development of MPNs and family history, Jewish descent, certain occupations, chemical exposures, certain types of residence, autoimmune medical conditions, blood donation, smoking, and obesity. Genetic patterns have also suggested inherited variants may predispose some populations to development of MPN and specific inherited genetic variation to development of MPN in rare families. A case-control study methodology has been developed to explore the epidemiology of MPNs further. Although this is a pilot study designed to develop the methodology it has had significant positive results. An association between main occupation of the household reflecting lower socio-economic status and MPN was shown. Current cigarette smoking was associated with PV and obesity with ET. A large multi-centre case-controlled study with collection of biological samples for molecular studies is in progress. This exciting study, which will be substantially larger than any previous work, should provide further insights into the epidemiology of MPNs.

## Figures and Tables

**Table 1:** Genetic predispositions associated with familial myeloproliferative neoplasms in population and individual families

Population Predisposition	Germline Variants in Families
*JAK*2 46/1 haplotype rs59384377 [23]	Germline duplication of *ATG2B* and *GSKIP* [24]
*TERT* gene germline variant rs2736100_C) [25,26]	*RBBP6*-R1569H [27]
Polymorphisms: *MECOM* rs2201862, *HBS1L-MYB* rs9376092, and *THRB-RARB* rs4858647 [28]	Novel variants in *LRRC3* and *BCORL1* [29]

**Table 2:** Environmental, lifestyle, and familial/ethic factors associated with myeloproliferative neoplasms

Factor	Significant Associations [10]
Family history	Relatives with female probands
Jewish decent	Ashkenazi Jewish descent [32,33]
Occupation	Poultry workers
	Commercial pressmen
	Petroleum refinery workers [34,35]
	Agricultural workers
	Rural sector workers
	Cooks and waiters
	Clerks
	Working with electrical devices
Chemical exposure	Benzene
	Petroleum
	Dark hair dye use for more than 10 years and ET [36]
Residence	Rural residence [37]
	Tuff house and ET [36]
Medical conditions	Crohn’s disease [38]
	Giant cell arteritis [39]
Blood donation	Southern Stockholm [40] France
Smoking	Increased risk of MPN in smokers compared to never smokers [41]
Body mass index	Increased risk of MPN with each 10 kg/m^2^ increase in body mass index [42]
Ionising radiation	Different pattern of driver mutations in those exposed to ionising radiation at Chernobyl [43]
